# Supersonic Dislocation Bursts in Silicon

**DOI:** 10.1038/srep26977

**Published:** 2016-06-06

**Authors:** E. N. Hahn, S. Zhao, E. M. Bringa, M. A. Meyers

**Affiliations:** 1 Materials Science and Engineering Program, University of California, San Diego, La Jolla, CA 92093, USA; 2 Ciencias Exactas y Naturales, Universidad Nacional de Cuyo, Mendoza 5500, Argentina; 3 CONICET, Mendoza 5500, Argentina

## Abstract

Dislocations are the primary agents of permanent deformation in crystalline solids. Since the theoretical prediction of supersonic dislocations over half a century ago, there is a dearth of experimental evidence supporting their existence. Here we use non-equilibrium molecular dynamics simulations of shocked silicon to reveal transient supersonic partial dislocation motion at approximately 15 km/s, faster than any previous *in-silico* observation. Homogeneous dislocation nucleation occurs near the shock front and supersonic dislocation motion lasts just fractions of picoseconds before the dislocations catch the shock front and decelerate back to the elastic wave speed. Applying a modified analytical equation for dislocation evolution we successfully predict a dislocation density of 1.5 × 10^12^ cm^−2^ within the shocked volume, in agreement with the present simulations and realistic in regards to prior and on-going recovery experiments in silicon.

The response of crystalline solids to shock compression is known to produce a variety of deformation mechanisms including dislocation nucleation and motion, twinning, phase transformations, and amorphization^1^,^2^. It is well established that each of these deformation modes is affected by strain-rate dependent effects. Extreme dislocation velocities have been theoretically predicted by Frank and van der Merwe^3^, Eshelby^4^, and Weertman^5^, but heretofore have not been observed experimentally. Dislocation velocities are classified into subsonic, transonic and supersonic depending on whether they are lower or exceed the shear or longitudinal wave velocity respectively. Thus far, only few molecular dynamics studies have confirmed their existence.

Molecular dynamics (MD) simulations by Gumbsch and Gao^6^ were first able to demonstrate stable motion of dislocations in tungsten with both transonic and supersonic velocities. In order to achieve these velocities, dislocations were created “at speed” under high shear stress. This was accomplished by applying a large simple shear to perfect single crystals. Li and Shi^7^ were able to show that speeds in excess of the shear wave velocity could be reached from stationary edge dislocation configurations. Simulations of aluminum by Vandersall and Wirth^8^ identify the short-lived stability of supersonic dislocations before deceleration to the elastic wave speed on picosecond timescales. Vandersall and Wirth^8^ also identify the formation of nano-twins. Ruestes *et al.*^9^ detail a compelling methodology that may be used to indirectly observe dislocation velocities by nano-indentation followed by a short duration laser shock pulse; this strategy was effective in simulations of tantalum, but the physical experiment remains inconclusive.

Modern experiments in copper using femtosecond detection of lattice dynamics have verified the high elastic limits predicted by MD, with elastic strains of 12–20% and dislocation densities that increase by two orders of magnitude during laser shock compression^10^. Calculations of mobile dislocation densities using Orowan’s equation importantly draw values of dislocation velocities directly from MD simulations on the same length and time scales^11^.

Regarding silicon, there are several shock experiments at applicable strain rates^2^,^12^,^13^,^14^ in addition to multiple MD simulations^2^,^13^,^14^,^15^,^16^. Notably, Smith *et al.*^17^ used laser-driven shocks to measure the elastic limit as a function of strain rate. For [001] single crystals at a calculated strain rate of 10^8^ s^−1^, the Hugoniot Elastic Limit (HEL) was measured to be 19 ± 3 GPa. The Gilman model including dislocation production was employed to explain their observed plastic relaxation rates and they fit their results using:


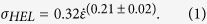


Thus, for 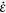 = 1 × 10^9^ s^−1^ as may be achieved during laser shock experiments, and 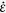 = 1 × 10^10^ s^−1^, as realized during atomistic shock simulations, the HEL is projected to lie between the wide ranges of 16–38 GPa and 25–64 GPa, respectively. This may explain results by Kalantar and co-workers^18^, where no plastic relaxation was observed by dynamic diffraction of silicon shocked to pressures somewhat lower than current estimates for the elastic limit. Absence of the diamond-cubic to β-Sn phase transition at strain rates of 10^10^ s^−1^ is also expected due to kinetic suppression as evidenced by experiments by Loveridge *et al.*^19^ up to 20 GPa at 10^6^ s^−1^ with no observable phase change.

For MD simulations, Oleynik *et al.*^20^ carried out large-scale shock simulations, showing that the Stillinger-Weber^21^ (SW) potential provided stress-strain curves for the diamond-cubic structure that compared well with *ab-initio* results when the strain is below 15–20%, corresponding to shear stresses below 7.5 GPa. Importantly, there have been a number of studies utilizing the SW potential to accurately model dislocations in silicon^22^,^23^,^24^.

## Homogenously nucleated supersonic dislocations

**Figure 1:**
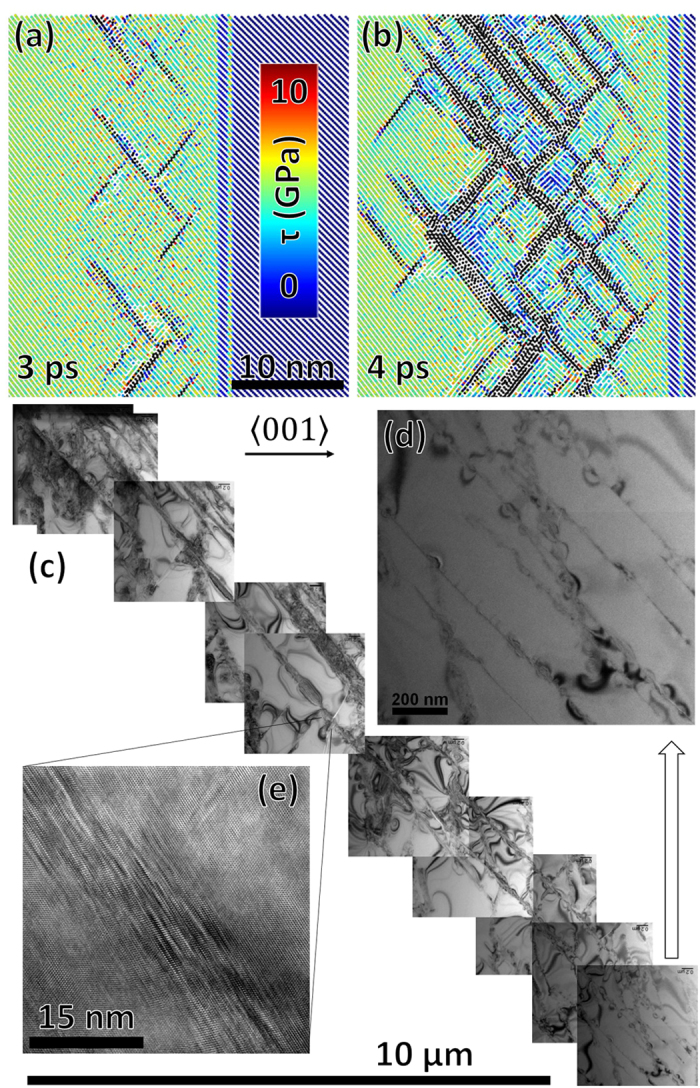
(**a**) Homogenous nucleation of stacking faults on {111} slip planes occurring at σ_z_ = 32.5 GPa and τ = 6.4 GPa as a shock wave travels from left to right. Atomic color is indicative of the absolute value of the local shear stress. (**b**) Significant relaxation (blue color) is seen in the 4 ps time step. (**c**) Recovered microstructure from a 50 J laser-driven shock experiment: ~11 GPa peak shock pressure^2^,^13^. (**d,e**) Higher magnifications showing the tip of the dislocation structure and a large mass of stacking faults, respectively. The growth of subsequent stacking fault layers can be seen in both molecular dynamics and laser experiments; this process occurs in order to expedite the relaxation of high shear stresses.

We observe, as postulated by Meyers^25^, homogenous nucleation of partial dislocations at the shock front; in silicon this occurs at a particle velocity (U_p_) = 1.9 km/s corresponding to a normal shock stress (σ_z_) of 31 GPa. Figure 1 shows multiple time snapshots for a shock of U_p_ = 2 km/s, for which, σ_z_ = 32.5 GPa and the shear stress is 6.4 GPa. At t = 4 ps, Fig. 1(b) stacking faults continue to nucleate homogeneously at the shock front. The motion of previously nucleated partials and heterogeneous nucleation of subsequent stacking-fault layers further behind the shock front are also observed. The simulations are in agreement with our experimental observations, by transmission electron microscopy, of stacking-fault generation in laser shock compressed silicon, as shown in Fig. 1(c–e). Figure 1(d) shows the detail of the tip of the dislocation array. Figure 1(e) shows a higher resolution image of an assembly of parallel stacking faults that are generated in proximity to the first one to relax the shear stresses. This is also simulated by MD and can be best seen by comparing Fig. 1(b,e).

**Figure 2:**
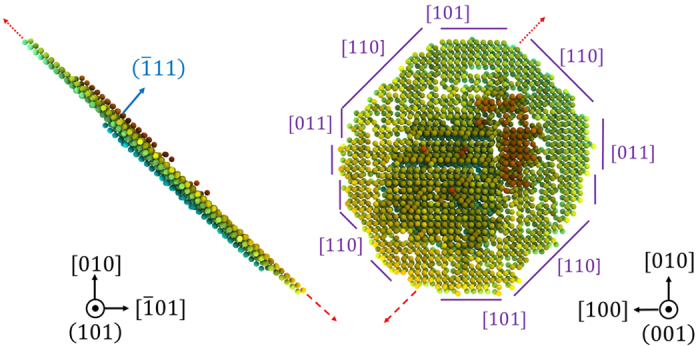
Dual projection view of stacking faults at 3 ps showing {111} slip plane and octagonal shear loop resulting from anisotropy of propagation direction. Note that the Burgers vector is conserved in a shear loop; the red arrow corresponds to the principal direction of shear loop motion inclined to the shock front.

The homogeneous nucleation threshold at a strain rate of 1 × 10^10^ s^−1^ is consistent with the lower range of the HEL extrapolated from the experimental results of Smith *et al.*^17^. Just as in other fcc-type systems, partial dislocations are nucleated in adjacent planes in order to more effectively relieve shear stress^8^. Figure 2 shows two projections of an isolated stacking fault selected from the 3 ps frame in Fig. 1. Adjacent stacking faults can be identified above and below the primary stacking fault. Red arrows indicate the direction of motion of the fastest moving partial dislocations. The separation distance along this direction determines one measure of the stacking-fault length used in subsequent calculations of dislocation velocity and dislocation density.

**Figure 3:**
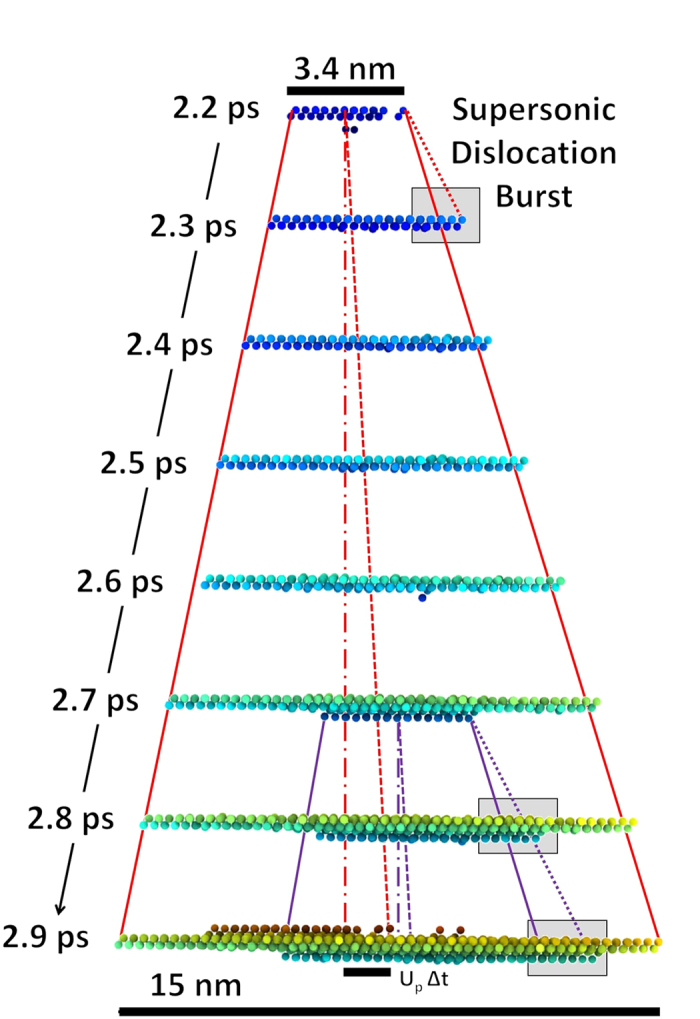
Time sequence from 2.2 to 2.9 ps showing partial dislocation velocity burst at 2.2 ps and velocity burst of secondary partial between 2.7 and 2.9 ps. Solid lines indicate motion at ~*U*_*L*_, dashed lines represent motion attributed for non-zero center of mass velocity and thus a non-stationary reference point, and dotted lines represent supersonic velocities. Gray areas indicate the supersonic bursts.

Figure 2 illustrates that the present observation is not simply a pair of equal and opposite partial dislocations, but a defect with correlated motion in multiple directions relative to the shock front-a case distinct from simple shear and of direct relevance to current shock experiments. Figure 3 gives the time evolution, from 2.2 to 2.9 ps, of a projection rotated from Fig. 2(a). In this projection, defect motion occurs on the “horizontal” 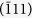 plane. A non-zero particle velocity implies a moving center of mass, taken here as αU_p_, where α accounts for the relationship between the shock direction and the direction of maximum dislocation velocity. This motion is visually represented in Fig. 3 as a dashed line compared to a stationary dot-dashed reference line. The stacking fault is shown to grow both towards (right) and away (left) from the shock front in Fig. 3. The slope corresponding to the velocity is indicated by solid lines. The evolution of dislocations/stacking faults can be visualized by the supplementary video.

At t = 2.2 ps, the velocity of the leading partial dislocation bursts to 12 km/s for approximately 0.1 ps before subsequently slowing down to 8.2 km/s, matching the velocity of the rear moving partial dislocation. For comparison, the elastic precursor to the shock front travels at a velocity U_s_ = 8.7 km/s.

Shear (transverse) and longitudinal wave speeds can be expressed as a function of pressure dependent fourth-order elastic moduli and density^26^. The shear wave speed in the <110> direction of propagation is anisotropic (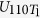
and 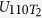). One of the anisotropic wave speeds is equivalent to the shear wave speed in the <001> direction; this wave speed is the larger of the two speeds and is used throughout the text as U_*T*_. Thus:


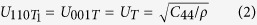



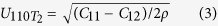


The longitudinal wave speeds referenced for the elastic wave speeds in the shock direction (U_*L*_) and the direction of dislocation motion [for 001] and [110] are:


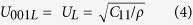



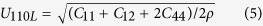


In accordance with Weertman^5^, we define the subsonic (*v*_*sub*_), transonic (*v*_*trans*_), and supersonic (*v*_*super*_) velocity regimes as follows: *v*_*sub*_ < U_*T*_, U_*T*_* < v*_*trans*_ < U_L_, and *v*_*super*_ > U_L_.

Table 1 shows C_*ij*_, density, two longitudinal velocities, and the larger transverse velocity along the <110> direction as calculated at equilibrium and at pressure using the Stillinger Weber potential. The supplemental material contains details for the relationship between the shock and hydrostatic pressure.

The simulation shows that, at t = 2.7 ps, a secondary set of partial dislocations is nucleated underneath the first stacking fault in order to further relieve shear stresses. The partial dislocation quickly accelerates and reaches a transient supersonic velocity of ~15 km/s for ~0.2 ps before decelerating due to interaction with surrounding/adjacent partial dislocations. At the high strain-rate elastic-plastic limit for silicon the shear stress is ~6 GPa and is near the theoretical shear strength of 6.5 GPa^27^. It is not surprising then that the motion of dislocations in this regime is highly transitory in nature.

### Dislocation density evolution

In 1958, Smith^28^ proposed a shock front interface composed of supersonic dislocations. The successive homogenous nucleation of dislocations at the shock front was used analytically by Meyers *et al.*^29^ for Cu to obtain a dislocation density. Here we adapt the analytical description incorporating a strain-rate dependent HEL term in addition to supersonic dislocation motion (complete derivation contained within the Supplemental Material). The dislocation density in the shocked volume is:


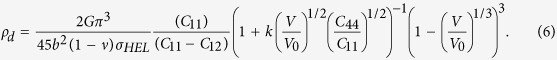


**Figure 4:**
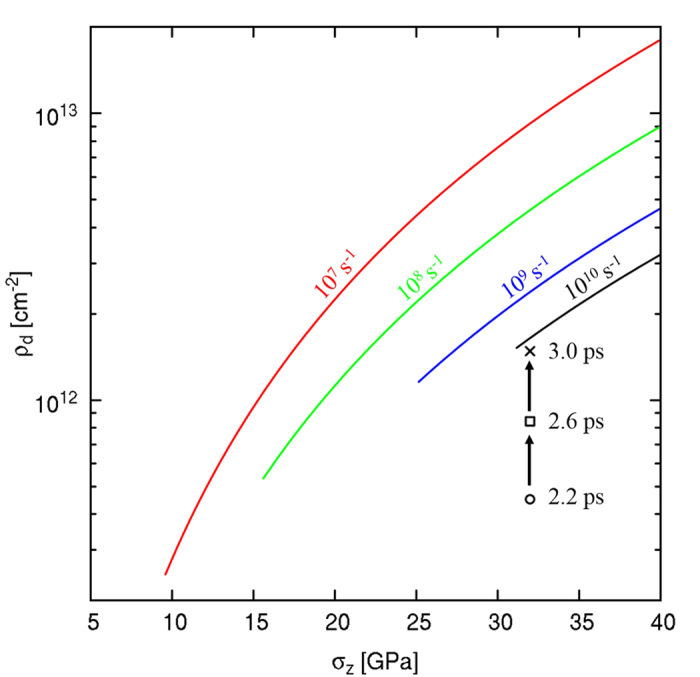
Analytical dislocation density prediction where each curve represents a unique strain rate. The dislocation density is highly dependent on the HEL and curves are truncated below this limit corresponding to an elastic response; the material would have a dislocation density unchanged from its intrinsic value. The symbols track the dislocation density evolution observed during the simulation. At 3.0 ps, the MD dislocation density approaches the one analytically predicted for 10^10^ s^−1^, demonstrating the agreement between the two approaches (analytical and MD).

The number of dislocations and the average partial separation can be extracted from the simulation (detailed further in the Supplemental Material). The average partial separation is 1.46 nm at 2.2 ps and reaches a steady state value of 3.12 nm at 3 ps. Taking the dislocation line length for *n* total stacking faults over the shocked volume we obtain an estimate of dislocation density (*ρ*_*d*_ = *l*_*d*_/V) plotted against the shock stress in Fig. 4. At nucleation, the dislocation density is 4.5 × 10^11^ cm^−2^ and evolves to reach a steady-state value of 1.5 × 10^12^ cm^−2^ at 3.0 ps. Each curve in Fig. 4 represents a different strain rate and thus a distinct HEL and a unique dislocation density curve calculated analytically from Eq. 6. As no dislocation production occurs during the elastic regime, each curve is truncated below the respective HEL.

Simulations of silicon under shock conditions directly comparable to laser experiments show homogenous dislocation nucleation and supersonic dislocation motion. The motion of dislocations proceeds supersonically (greater than the longitudinal sound speed in their respective direction of motion), but such speeds are highly transient as shear stress is quickly relaxed and dislocations catch up to the shock front. The present calculations are able to distinguish the difference between transonic and supersonic dislocations due to the significant overshoot of the supersonic velocity. Near the exact transition it is difficult to determine whether the dislocation travels exactly at or slightly below the wave speed due to the coupled relations between dislocation motion, shear relaxation, and local elastic moduli.

Corresponding to a Hugoniot elastic limit of 31 GPa, the model predicts a dislocation density of 1.48 × 10^12^ cm^−2^. This is in excellent agreement with steady state dislocation densities extracted directly from the present simulations (4.5 × 10^11^ −1.5 × 10^12^ cm^−2^). Post-shock observations in monocrystalline Si show that partial dislocations travel uninterruptedly for distances of over 10 μm; this is indirect evidence that these dislocations, nucleated close to the surface, track the shock front for long distances and thus relax the high shear stresses that would otherwise generate new dislocation arrays. These findings have critical ramifications in evaluating the densities of dislocations from shock experiments^2^,^11^ and offer a direct explanation for high dislocation content commonly utilized in plastic relaxation models^17^.

## Methods

### Molecular Dynamics Simulations

Elastic and plastic deformation of silicon in the strain rate regime of 10^9^ – 10^10^ s^−1^ is investigated by large-scale, non-equilibrium molecular dynamics simulations consisting of 7.5 million atoms (dimensions 50a_o_ × 50a_o_ × 300a_o_, a_o_ = 0.543 nm). Single crystal samples simulated with the Stillinger Weber potential^21^ were thermalized at 300 K and periodic boundary conditions are applied transverse to the shock direction. In order to study the homogenous nucleation of defects at the shock front, a frozen piston (a few atomic layers thick and in perfect contact with the target) undergoes a linear ramp to the desired particle velocity over 2 ps. This methodology eliminates loading of surfaces and/or interfaces which prompt heterogeneous dislocation/twin nucleation at surface steps^22^ or reconstructed dimers. This setup mimics the initial stages of laser-shock experiments that produce principally uniaxial strain during loading^17^. OVITO^30^ is utilized to visualize and render the atomic systems.

### Laser Experiments

Laser driven shock-recovery experiments were conducted at Omega laser facility, Laboratory of Laser Energetic, University of Rochester. The pulsed laser, with a FWHM duration of 1 ns and nominal energy of 50 J, was focused to a spot size of 3 mm, yielding a laser intensity of around 10^11^*W*/cm^2^. Such high density energy, once deposited onto the target, will ablate its surface and create a shock wave that propagates through the target; the initial pressure is ~11 GPa. Due to the extremely short laser duration, the shock pulse will decay rapidly, yielding an extremely high strain rate (10^7^~10^9^ s^−1^) that is inaccessible by any other techniques and comparable to our MD simulations. The ultrafast loading rates also prevent the catastrophic failure of materials by massive crack coalescence, thus enabling the successful recovery of brittle silicon target from high pressure/shear experiments.

## Additional Information

**How to cite this article**: Hahn, E. N. *et al.* Supersonic Dislocation Bursts in Silicon. *Sci. Rep.* **6**, 26977; doi: 10.1038/srep26977 (2016).

## Supplementary Material

Supplementary Information

Supplementary Video

## Figures and Tables

**Table 1:** Pressure dependent second order elastic moduli and wave speeds.

σ_z_*(GPa)*	P *(GPa)*	*C*_11_ *(GPa)*	*C*_12_ *(GPa)*	*C*_44_ *(GPa)*	*ρ (g/cm*^*3*^)	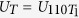 *(km/s)*	*U*_*L* <*100*>_*(km/s)*	*U*_*L* <*110*>_*(km/s)*
0.0	0.0	151.4	76.4	56.4	2.32	4.93	8.08	11.54
16.5	12.0	184.8	108.8	57.8	2.54	4.77	8.53	12.92
32.5	26.5	197.9	136.2	44.0	2.73	4.01	8.51	13.44

Note that the partial dislocation accelerates and reaches a transient supersonic velocity of ~15 km/s for ~0.2 ps.
