# Supplementary material for: Supersonic Dislocation Bursts in Silicon
Source: Sci Rep. 2016 Jun 6;6:26977. doi: 10.1038/srep26977 (PMC4893603; doi:10.1038/srep26977)
Supplement: Supplementary Information [file srep26977-s1.pdf]

Supersonic Dislocation Bursts in Silicon: Supplemental Material

E. N. Hahn[1], S. Zhao[1], E. M. Bringa[2,3], M.A. Meyers[1*]

[1]Materials Science and Engineering Program, University of California, San Diego, La Jolla, CA 92093, USA

[2]Ciencias Exactas y Naturales, Universidad Nacional de Cuyo, Mendoza 5500, Argentina

[3]CONICET, Mendoza 5500, Argentina

[*]corresponding author: mameyers@eng.ucsd.edu

Dislocation Density Derivation

In 1958, Smith[1] proposed a shock front interface composed of supersonic dislocations. However, it did not predict a dislocation density increase due to shock which was resolved by sequential homogenous nucleation of dislocations at the shock front. This was calculated analytically by Meyers et al.[2–4] for Cu.

Here we adapt the analytical description developed by Meyers and leave in a strain-rate dependent HEL in addition to an increased separation of nucleation separation due to supersonic dislocation motion. Dislocation density can be defined as the inverse separation of dislocations in two dimensions, lateral to the shock front, *d*, and aligned with the shock front, h_s. Accounting for two dislocations per stacking fault, the following principal relationship for dislocation density can be written as:

$$\rho_d = \left(\frac{dh_s}{2}\right)^{-1}. \tag{1}$$

Taking a standard expression for lateral separation of dislocations (*d*) in order to account for a given strain, ε, the following expressions can be written in terms of original and shocked lattice parameter (a_0 and a_s respectively):

$$d = \frac{a_0}{\varepsilon}, \tag{2}$$

$$\varepsilon = \frac{a_0 - a_s}{a_0}. \tag{3}$$

The separation of dislocations can then be expressed in terms of lattice parameter,

$$d = \frac{{a_0}^2}{a_0 - a_s}. \tag{4}$$

The inverse of lateral separation can be manipulated into the following form:

$$d^{-1} = \frac{1}{a_0} - \frac{1}{a_0}\frac{a_s}{a_0}. \tag{5}$$

Taking a typical relationship between Burgers vector, b, and lattice parameter,

$$a_0 = b\sqrt{2}\,, \tag{6}$$

in addition to a relationship between instantaneous volume, V, and equilibrium volume, V_0, as related to the shock and equilibrium lattice parameter,

$$\left(\frac{a_s^{\,3}}{a_0^{\,3}}\right) = \frac{V}{V_0}\,. \tag{7}$$

We can now write an expression for d^{-1} in terms of only one variable, V:

$$d^{-1} = \frac{1}{b\sqrt{2}}\left(1-\left(\frac{V}{V_0}\right)^{1/3}\right), \tag{8}$$

Following formalism previously developed[2], the contribution of the stress field from each dislocation only produces stress in the σ_{12} component of the stress tensor. The sum of these contributions for a spacing of $1/n^2$ dislocations extending infinitely along a planar shock front is equal to $\pi^4/90$ and thus the expression for stress in terms of subsequent emission distances, h, can be written as:

$$\sigma_{12} = \frac{Gb}{2\pi(1-\nu)}\frac{2\sqrt{2}}{n^2 d^2}h = \frac{Gb\pi^3 h}{45\sqrt{2}(1-\nu)d^2}\,, \tag{9}$$

The critical shear stress, σ_{12}^c, required to nucleate a dislocation can be defined through the HEL:

$$\frac{\sigma_{12}^{\;c}}{\sigma_{HEL}} = \frac{(C_{11}-C_{12})}{2(C_{11})}\,, \tag{10}$$

$$\sigma_{12}^{\;c} = \frac{\sigma_{HEL}(C_{11}-C_{12})}{2(C_{11})}\,, \tag{11}$$

Setting $\sigma_{12} = \sigma_{12}^c$, the separation of subsequent partial dislocation nucleation sites is:

$$h = \frac{45(1-\nu)}{Gb\pi^3\sqrt{2}}\frac{(C_{11}-C_{12})}{2(C_{11})}\sigma_{HEL}d^2\,. \tag{12}$$

The effective separation, h_s, is then increased when accounting for mobile dislocations at the shock front which aid in relaxation:

$$h_s = h\left(1+\frac{kv_d}{U_s}\right). \tag{13}$$

Here, k is an orientation factor equal to 1 for <001> shocks in fcc or diamond cubic materials. The shock speed, U_s, is well defined as:

$$U_s = \left(\frac{E_L}{\rho} \right)^{1/2} . \quad (14)$$

While the minimum supersonic dislocation velocity relative to the shock front, v_d, is defined as:

$$v_d = \sqrt{2} \frac{\sqrt{2}}{2} C_s = \left(\frac{E_T}{\rho} \right)^{1/2} . \quad (15)$$

The effective shock separation can now be written as:

$$h_s = h \left(1 + k \left(\frac{\rho_0}{\rho} \right)^{1/2} \left(\frac{1-2v}{1-v} \right)^{1/2} \right) = h \left(1 + k \left(\frac{V}{V_0} \right)^{1/2} \left(\frac{C_{44}}{C_{11}} \right)^{1/2} \right). \quad (16)$$

Substituting eq. (16) into eq. (1) gives:

$$\rho_d = \frac{2}{d^3} \frac{Gb\pi^3\sqrt{2}}{45(1-\nu)} \frac{2(C_{11})}{(C_{11}-C_{12})} \frac{1}{\sigma_{HEL}} \left(1 + k \left(\frac{V}{V_0} \right)^{1/2} \left(\frac{C_{44}}{C_{11}} \right)^{1/2} \right)^{-1} , \quad (17)$$

Substituting eq. (8) into eq. (17) now gives a final expression for dislocation density due to homogenous dislocation nucleation at the shock front:

$$\rho_d = \frac{2G\pi^3}{45b^2(1-\nu)\sigma_{HEL}} \frac{(C_{11})}{(C_{11}-C_{12})} \left(1 + k \left(\frac{V}{V_0} \right)^{1/2} \left(\frac{C_{44}}{C_{11}} \right)^{1/2} \right)^{-1} \left(1 - \left(\frac{V}{V_0} \right)^{1/3} \right)^3 . \quad (18)$$

Partial Dislocation Identification

In order to identify partial dislocations in the system, the primary identification scheme utilized a coordination based evaluation. We employ a cutoff radius of 3.0 Å which captures the addition of "new neighbors" during a partial displacement shuffle. This cutoff value must be below the expected neighbor distances in many polymorphs of silicon (3.2 Å) as well as the equilibrium distance of the second neighbor shell (~3.8 Å) which may significantly decrease during compression. Notably, this distance also corresponds to the bond distance between silicon atoms in many amorphous and 5 coordinated structures. We show below a comparison of analysis techniques (Fig S1) and a snapshot of partial identification near the shock front (Fig S2).

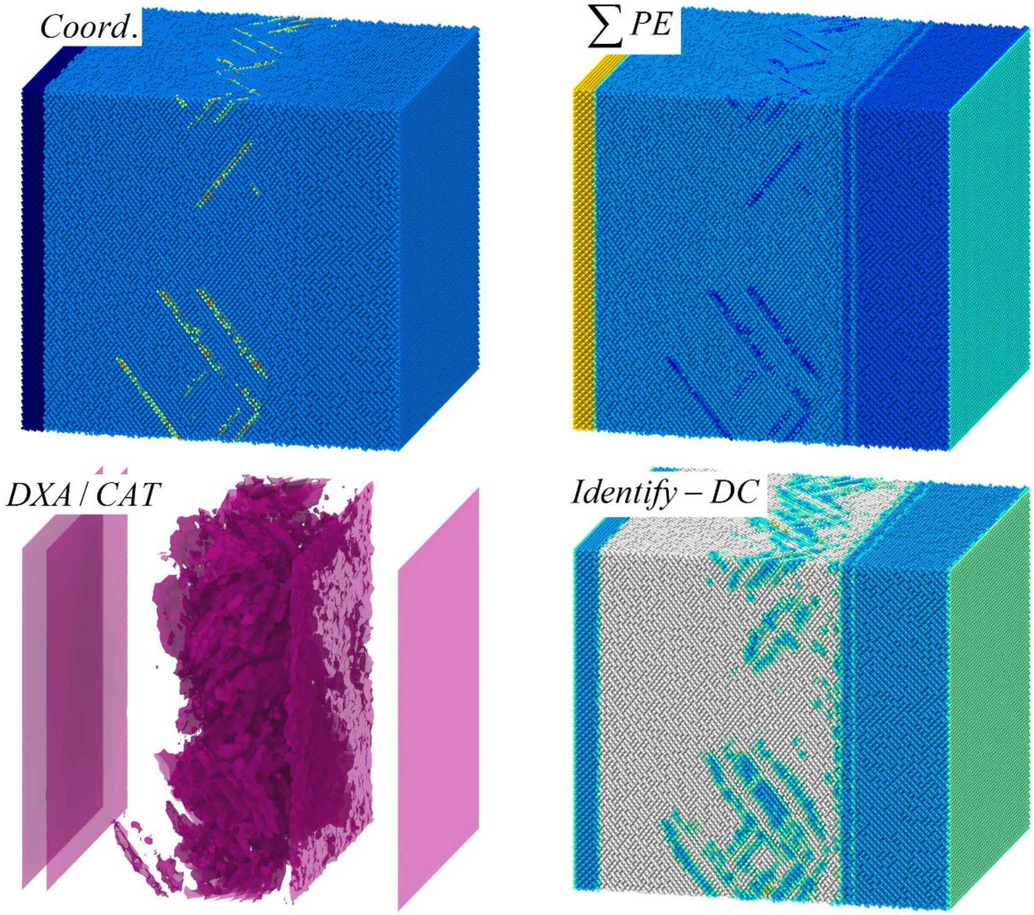

Figure S1. Stacking fault identification schemes. Coordination with r_c = 3 Å (Top Left), Potential energy (Top Right), Dislocation Extraction Algorithm (Bottom Left)[5], and Identify Diamond Structure (Bottom Right)[6].

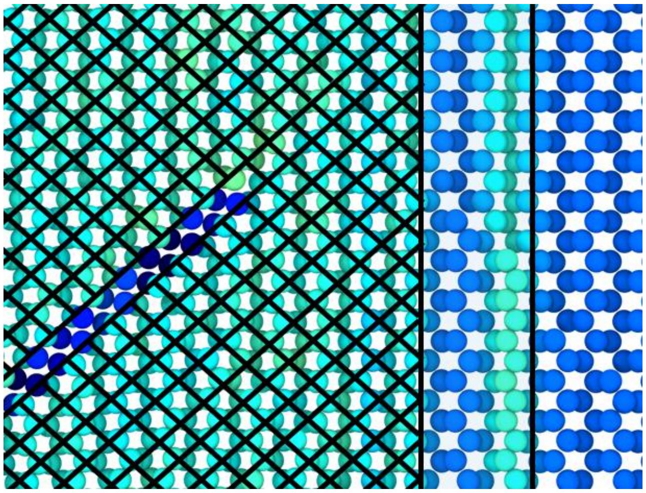

Figure S2. Identification of partial dislocation near shock front by potential energy criterion. The partial dislocation tip is identified by the furthest dark blue atom corresponding to a shift in the lattice across the stacking fault.

Dislocation Density Calculation from Simulation

In order to extract an accurate dislocation density from the simulation, OVITO's surface identification tool[7] was used to relate surface area and volume of defective atoms. When defects have the thickness of only a single stacking fault they can be considered as thin disks of thickness, *t*. The relationship between surface area and volume, $R_{S/V}$, provides an expression for the circumference, and thus the partial separation, s_p, and the dislocation line length, l_d :

$$R_{S/V} = \frac{\pi s_p t + \frac{2}{4}\pi s_p{}^2}{\frac{1}{4}\pi s_p{}^2 t} = 4s_p{}^{-1} + 2t^{-1}, \quad (19)$$

$$s_p = 4(R_{S/V} - 2t^{-1})^{-1}, \quad (20)$$

$$l_d = \pi s_p. \quad (21)$$

Relationship between uniaxial and hydrostatic stress.

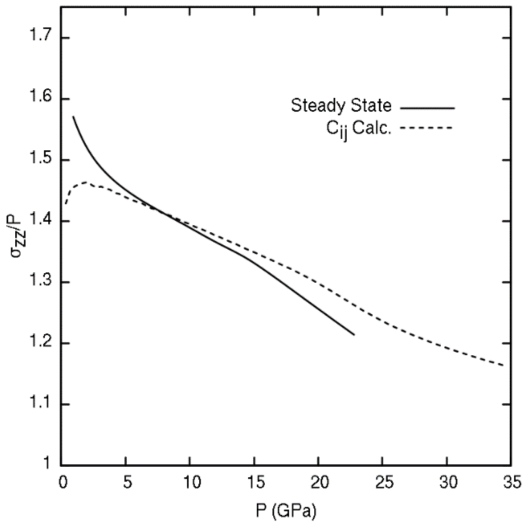

Figure S3. Relationship between "zz" component of the stress tensor and hydrostatic pressure compared during a steady state shock (created through a ramp) and C_{ij} calculations for a system undergoing uniaxial compression.

The approximate relationship we identify for the <001> direction is $\sigma_z = 1.49(P) - 0.01(P)^2$.

Supplementary Video: Homogeneous Nucleation of Dislocations at Shock Front in Silicon

The video contains the time evolution of homogenously nucleated partial dislocations at the shock front and their subsequent motion towards and away from the moving shock front. The time period featured

in the video is between 2 and 4 ps; this directly corresponds to the U_p = 2 km/s simulation as presented in the main text (Figs. 1-4).

References

1. Smith, C. S. Metallographic Studies of Metals After Explosive Shock. *Trans Met Soc AIME* **Vol: 212,** (1958).
2. Meyers, M. A., Jarmakani, H., Bringa, E. M. & Remington, B. A. in *Dislocations in Solids* (ed. Kubin, J. P. H. and L.) **15,** 91–197 (Elsevier, 2009).
3. Meyers, M. A. *et al.* Laser-induced shock compression of monocrystalline copper: characterization and analysis. *Acta Mater.* **51,** 1211–1228 (2003).
4. Meyers, M. A. A mechanism for dislocation generation in shock-wave deformation. *Scr. Metall.* **12,** 21–26 (1978).
5. Stukowski, A. & Albe, K. Extracting dislocations and non-dislocation crystal defects from atomistic simulation data. *Model. Simul. Mater. Sci. Eng.* **18,** 085001 (2010).
6. Maras, E., Trushin, O., Stukowski, A., Ala-Nissila, T. & Jonsson, H. Global transition path search for dislocation formation in Ge on Si(001). *ArXiv160106597 Cond-Mat* (2016).
7. Stukowski, A. Computational Analysis Methods in Atomistic Modeling of Crystals. *JOM* **66,** 399–407 (2013).
